# Education level and incident functional disability in elderly Japanese: The Ohsaki Cohort 2006 study

**DOI:** 10.1371/journal.pone.0213386

**Published:** 2019-03-12

**Authors:** Dieta Nurrika, Shu Zhang, Yasutake Tomata, Yumi Sugawara, Fumiya Tanji, Ichiro Tsuji

**Affiliations:** 1 Department of Health Informatics and Public Health, Division of Epidemiology, Tohoku University School of Public Health, Graduate School of Medicine, Sendai, Miyagi, Japan; 2 Banten School of Health Science, Ministry of Research, Technology and Higher Education, Higher Education Service Institutions (LL-DIKTI) Region IV, Bandung, West Java, Indonesia; University of Brescia, ITALY

## Abstract

As the factors that link education level with incident functional disability in elderly Japanese have never been investigated, the present study investigated this issue in an elderly Japanese population. A 9-year prospective cohort study (2006–2015) was conducted among 8,680 Japanese individuals (≥65 years), Ohsaki city, Japan. In a baseline survey, we collected data on education level and potential mediators. Data on incident functional disability were retrieved from the Long-term Care Insurance database. The Cox proportional hazards model was used to estimate the hazard ratios (HRs) and 95% confidence intervals (CIs) for incident functional disability by education level (below upper-secondary education (reference), and upper secondary education and above). Mediating effects were estimated using accelerated failure time model and a logistic regression model. During 9-year follow-up period, 2,742 cases (31.6%) of incident functional disability were observed, and education level showed an inverse association with functional disability (*P* for trend <0.01). Participation in community activities had the largest mediating effect (34.7%) on the relationship between education level and incident functional disability. This effect remained among those aged 65–74 years (19.9%) but became negligible among those aged ≥75 years. Other potential mediators (such as smoking and drinking status) were also tested, but these showed only small mediating effects. The inverse association between education level and the incident risk of functional disability appears to be largely mediated by participation in community activities among elderly Japanese, especially those aged 65–74 years.

## Introduction

The proportion of elderly individuals in the population of Japan is projected to increase rapidly. In 2015, the number of people aged 65 years and over was approximately 26%, and by 2036 and 2065 it is estimated that this proportion will be 33% and no less than 38%, respectively [1]. The concept of healthy aging is therefore becoming crucial for elderly Japanese and for Japanese society as a whole, and disability prevention is one aspect of this issue [2].

Education is an important factor related to functional disability [3]. Emerging evidence suggests that education level has a significant association with incident functional disability [4–9]. Previous studies in western countries have also suggested that certain mediators [10], including but not limited to body mass index (BMI), smoking status, drinking status, time spent walking, social support, and participation in the community [11–14], affect this association. Therefore, identifying the crucial mediators between education level and incident disability becomes a issue of great concern to disability prevention among Japanese elderly population.

To date, however, only one study has investigated mediating effect in association between education level and health outcome in terms of functional limitation in the middle-aged (50–59 years) Japanese population [15], and the findings suggested that social participation was the primary mediators.

Japanese elderly people have major causes of disability (top three: dementia, stroke, and frailty) that differ from those in middle-aged people (top three: stoke, joint disease, and spinal cord injury) [16], which suggests that different mediators and different mechanisms might operate in the education-mediator-disability paths. Furthermore, among the elderly population, the major causes of disability also vary between younger elderly (aged 65–74 years) (stroke) and older elderly (aged ≥75 years) (dementia), which may be attributed to differences in socioeconomic status and lifestyle behavior between the two age groups. This is because, in Japan, those aged 65–74 years are more likely to continue in paid employment than those aged 75 years and over [17]. For these reasons, we consider that mediating effects might differ according to age, even among the elderly.

The objective of the present cohort study was to investigate the association between education level and incident functional disability in an elderly Japanese population, and to clarify the factors mediating this association.

## Methods

### Study cohort

The data used for the present study were obtained from the Ohsaki Cohort 2006 study, the design of which has been described in detail elsewhere [18]. In short, the source population for the baseline survey comprised 31,694 men and women aged 65 years or older who were living in Ohsaki City, northeastern Japan, in December 2006.

The baseline survey was conducted to collect information on not only education level, but also history of diseases, body weight, height, smoking status, drinking status, time spent walking, participation in community activities, and social support [19].

**Fig 1:**
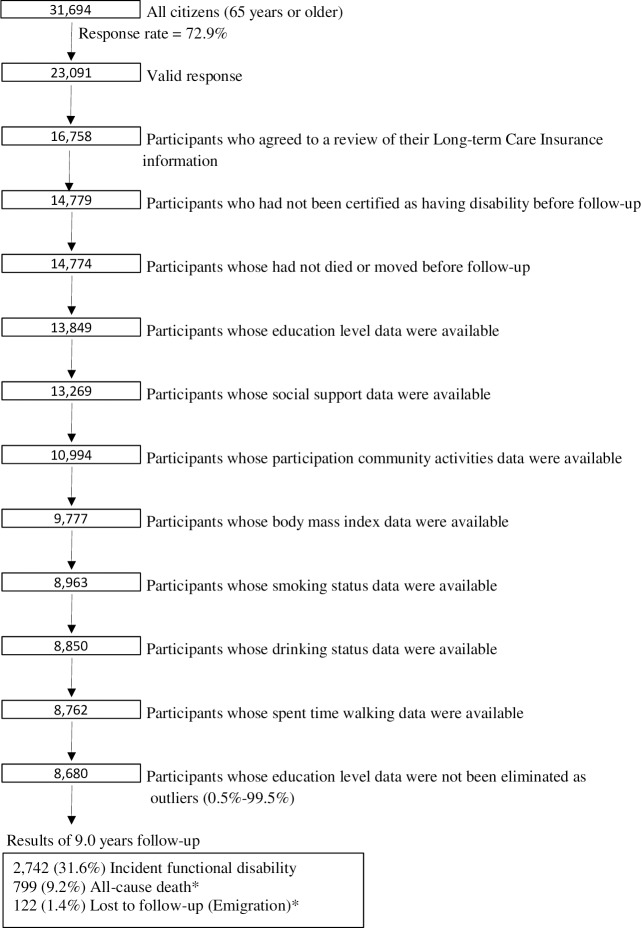
Flow chart of the study participants: The Ohsaki cohort 2006 study, Ohsaki city, Japan, 2006–2015. *without experiencing incident disability.

The baseline survey was conducted between 1 and 15 December 2006. A questionnaire was distributed by the heads of individual administrative districts to individual households and returned by post. Among the 31,694 participants, 23,091 who returned valid responses formed the study cohort **(Fig 1**).

Among eligible participants, we excluded 6,333 who did not provide written consent for review of their long-term care insurance (LTCI) information, 1,979 who had already been certified as having a disability by the LTCI at the time of the baseline survey, five who had died or moved out of the region during the period of the baseline survey, 925 whose education level data were missing, 5,087 whose data on potential mediators (including participation in community activities, social support, body mass index, smoking status, drinking status, and time spent walking; any of them) were missing, and 82 whose education level data lay outside the 0.5–99.5 percentile of the total range (<10 years and ≥30 years). This left 8,680 persons for inclusion in the present analysis.

This was a cohort study with a 9-year follow-up period from 16 December 2006 to 30 November 2015. Within this period, 122 persons were lost to follow-up because they migrated out of the study area without developing incident functional disability; accordingly, the follow-up rate was 98.6%. Among 62,374 person-years, incident functional disability was confirmed for 2,742 (31.6%) individuals, and the total number of all-cause deaths without incident functional disability was 799.

### Assessment of education level

Education level was assessed using the question “How old were you when you left school?” and answered using a positive integer. Based on the nine-year compulsory education system (age from 6 to 15 years) in Japan [20], all Japanese need to complete junior high school education (i.e. below upper-secondary education according to the International Standard Classification of Education, ISCED 2011 [21]). Additionally, the method used to calculate mediating effects requires the exposure to be classified into 2 categories [22, 23]. Therefore, we categorized education level into two groups: below upper-secondary education (<16 years) and upper secondary education and above (≥16 years).

### Assessment of mediators

Participation in community activities, social support, BMI, smoking status, drinking status, as well as time spent walking were treated as potential mediators in the relationship between education level and incident functional disability [11–14].

Social support was assessed by asking the following questions: “Do you have someone 1) with whom you can talk when you are in trouble; 2) whom you can consult when you do not feel well; 3) who can help you with your daily housework; 4) who can take you to a hospital when you feel ill; and 5) who can take care of you if you become bedridden?” to which respondents answered “yes” or “no” for each item. Then, we classified the respondents into two categories: 1) non-support (i.e., participants who perceived that they were did not have access to all 5 types of social support); 2) any form of social support (i.e., participants who perceived that they had access to at least one type of social support) [24].

We also assessed participation in community activities by asking how often the subjects participated in the following: 1) neighborhood associations; 2) sports or hobby groups, group entertainments; 3) volunteering for activities related to nonprofit organizations; and 4) participation in other types of social gathering. The frequency of each activity was assessed as never, a few times per year, 1 time/month, 2 to 3 times/month, 1 time/week, 2 to 3 times/week and ≥4 times/week [25]. Furthermore, we divided the participants into two groups: 1) non-participation (i.e., subjects who never participated in any of the four types of community activity), and 2) participation (i.e., subjects who participated in at least one type of community activity) [24].

BMI was calculated from self-reported body weight (in kilograms) divided by the square of body height (in meters), and then classed into two groups: <25.0 kg/m^2^; and ≥25.0 kg/m^2^. Smoking status and drinking status were classified as non-current (never and former) and current, respectively. Time spent walking was classified as: ≥1 hours/day, and <1 hour/day.

### The LTCI system in Japan

In this study, we defined incident functional disability as certification for LTCI in Japan. This is a mandatory form of social insurance for the frail elderly that was implemented in 2000. Every individual aged 40 years and older pay premiums, and everyone aged 65 years and older is eligible for formal caregiving services [26, 27]. When a person is judged to be eligible, the Municipal Certification Committee decides on one of seven levels of support, ranging from support level 1 to 2, and care level 1 to care level 5. The LTCI system has been described in detail elsewhere [28, 29].

### Follow-up (incident functional disability)

Incident functional disability was set as the endpoint, and this was defined as newly certified disability (support level 1 or higher) based on the LTCI system. The person-years of follow-up were counted for each subject from 16 December 2006 until the date of incident disability, date of emigration from Ohsaki city, date of death, or the end of the study period (30 November 2015), whichever occurred first.

We obtained a dataset that included information on LTCI certification, death or emigration from Ohsaki City. All data were transferred from the Ohsaki City government under an agreement related to Epidemiologic Research and Privacy Protection yearly each December.

### Ethical issues

The return of completed questionnaires was considered to imply consent to participate in the study involving the baseline survey data and subsequent follow-up of death and emigration. Information regarding LTCI certification status was confirmed after obtaining written consent returned from the participants at the time of the baseline survey. The Ethics Committee of Tohoku University Graduate School of Medicine reviewed and approved the study protocol.

### Statistical analyses

We counted the person-years of follow-up for each subject from 16 December 2006 until the date of incident functional disability, date of emigration from Ohsaki City, date of death, or the end of the study period (30 November 2015), whichever occurred first. In our analysis, deaths without LTCI certification were treated as censored.

Baseline characteristics were evaluated using analysis of variance for continuous variables and the chi-squared test for categorical variables. The Cox proportional hazards model was used to estimate the hazard ratios (HRs) and 95% confidence intervals (CIs) for incident functional disability according to education level, using group below upper-secondary education as a reference. The base model was adjusted for age (65–69, 70–74, 75–79, 80–84, or ≥85 years) and sex. This analysis was performed for all participants and two subgroups stratified by age (65˗74 years and ≥ 75 years).

In addition, sensitivity analysis was conducted to examine whether inclusion of participants with missing data for potential mediators would change the relationship between education level and incident functional disability.

To estimate mediating effects, we used a SAS macro [23] featuring 1) an accelerated failure time model for time to incident functional disability conditional on exposure (education), mediator, and confounders, and 2) a logistic regression model to which mediators conditional on exposure and confounders were fitted. Combining these estimates, the following effects were estimated: 1) any controlled direct effect (i.e. effect of education on incident functional disability while controlling the mediator at a fixed level); 2) any natural direct effect (i.e. effect of education on incident functional disability while setting the mediator at a level that would be observed in the reference level of education); 3) any natural indirect effect (i.e. effect of education on incident functional disability operating through a mediator level defined as that which would be observed in the reference level of education vs. a level that would be observed in the other level (i.e. outside the reference level) of education, while controlling education at the reference level); and 4) the total effect (i.e. overall effect of education level on incident functional disability), separately. Age (65–69, 70–74, 75–79, 80–84, or ≥85 years) and sex were controlled for in the models as confounding factors in education-mediator, education-disability, and mediator-disability relationships. Interactions between education level and mediators were also checked before mediating effects were assessed. If there was no interaction between education level and a mediator, the controlled direct effect would be equal to the natural direct effect (See S1 File. Appendix for more details). Thus the “proportion mediated” was calculated as the ratio of the natural indirect effect to the total effect. Mediators were categorized into dichotomous variables: BMI [<25 kg/m^2^ (reference) and ≥25.0 kg/m^2^], smoking status [current, and non-current (reference)], drinking status [current, and non-current (reference)], and time spent walking per day [<1 hour, and ≥1 hour (reference)], social support [(any form of social support (reference), and non-support] and participation in community activities [(participation in any community activity (reference), and non-participation]. Mediating effect of each mediator was estimated seperately by putting only one mediator into the model at a time.

Furthermore, the above analyses were re-conducted after stratifying for age (65–74 years and ≥75 years).

SAS statistical software package (version 9.4; SAS Institute Inc.) was used in all analyses, and all statistical tests were two-sided. Differences at P<0.05 were considered to be statistically significant.

## Results

### Baseline characteristics

**Table 1** shows the baseline characteristics of the participants overall according to education level. Participants with a higher education level were younger and less likely to be men, to be current smokers, to have time spent walking, and to have someone to take care of them. They were also more likely to participate in community activities.

**Table 1:** Baseline characteristics of the participants according to education level (n = 8,680). Ohsaki city, Japan, 2006–2015.

	Education level ^b^		*P-*value ^a^
	Below upper-secondary education	Upper-secondary education and above	
No. of all participants	2,452	6,228	
Age, y (mean±SD)	75.4±6.2	72.5±5.6	< 0.01
Sex, males (%)	56.2	48.3	< 0.01
History of diseases (%)			
Stroke	2.7	2.9	0.47
Hypertension	45.1	44.4	0.57
Myocardial Infarction	5.5	5.1	0.50
Diabetes	12.0	12.7	0.41
Cancer	9.0	10.1	0.14
Body Mass Index (mean±SD)	23.6±3.6	23.6±3.2	< 0.01
Current smoker (%)	15.5	13.0	< 0.01
Current alcohol drinker (%)	37.4	39.5	0.07
Time spent walking ≥ 1 h/d (%)	28.3	25.9	0.02
Participation in community activities (%)			
Activities in neighborhood association	38.5	51.7	< 0.01
Sports clubs, hobby groups	33.0	53.2	< 0.01
Volunteering	22.8	36.5	< 0.01
Social gathering	35.5	56.0	< 0.01
Social support (%)			
To consult when you are in trouble	90.1	90.2	0.93
To consult when you are in poor physical condition	93.8	93.7	0.80
To help with your daily housework	85.7	85.3	0.67
To take you to a hospital	93.3	92.1	0.06
To take care of you	88.6	86.7	0.02

^a^ Obtained by using a chi-square test for variables of proportion and 1-factor ANOVA for continuous variables.

^b^ Age at leaving school.

### Education level and incident functional disability

**Table 2** shows the relationship between education level and incident functional disability (n = 8,680). Compared to participants with education level below upper-secondary education, the HRs (95% CI) were 0.81 (0.75–0.88) for those with education level upper secondary education and above after adjustment for age and sex (*P* for trend <0.01). Similar results were also observed when stratified by age (65–74 years and ≥75 years; **Table 3** and **Table 4**, respectively).

**Table 2:** Relationship between education level and incident functional disability. Ohsaki city, Japan, 2006–2015.

	Education level ^b^		*P* for Trend
	Below upper-secondary education	Upper-secondary education and above	
No. of participants (n = 8,680)^a^	2,452	6,228	
Incident functional disability (%)	11.6	20	
Incident rate/1000 person years	62.9	37.4	
Crude	1.00 (reference)	0.58 (0.54–0.63)	< 0.01
Age ^c^ and sex adjusted	1.00 (reference)	0.81 (0.75–0.88)	< 0.01
No. of participants (participants with missing data for potential mediators were included, n = 13,849)^a^	4,140	9,709	
Incident functional disability (%)	12.8	21.7	
Incident rate/1000 person years	66.2	42.4	
Crude	1.00 (reference)	0.62 (0.59–0.66)	< 0.01
Age ^c^ and sex adjusted	1.00 (reference)	0.85 (0.80–0.90)	< 0.01

^a^ Analyses by cox proportional hazard model.

^b^ Age at leaving school.

^c^ Age (65–69, 70–74, 75–79, 80–84, or ≥85y).

**Table 3:** Relationship between education level and incident functional disability by age. Ohsaki city, Japan, 2006–2015 (n = 8,680)^a^.

	Education level^b^		*P* for Trend
	Below upper-secondary education	Upper-secondary education and above	
Age 65–74 years			
No. of participants (n = 5,338)	1,143	4,195	
Incident functional disability (%)	5.3	12.6	
Incident rate/1000 person years	32.8	19.8	
Crude	1.00 (reference)	0.59 (0.51–0.68)	< 0.01
Age^c^ and sex adjusted	1.00 (reference)	0.62 (0.54–0.72)	< 0.01
Age≥75 years			
No. of participants (n = 3,342)	1,309	2,033	
Incident functional disability (%)	21.6	31.8	
Incident rate/1000 person years	98.5	85.3	
Crude	1.00 (reference)	0.85 (0.77–0.94)	< 0.01
Age^d^ and sex adjusted	1.00 (reference)	0.91 (0.83–1.00)	0.06

^a^ Analyses by cox proportional hazard model.

^b^ Age at leaving school.

^c^ Age (65–69, 70-74y).

^d^ Age (75–79, 80–84, or ≥85y).

**Table 4:** Relationship between education level and incident functional disability by age. Ohsaki city, Japan, 2006–2015 (n = 13,849)^a^.

	Education level^b^		*P* for Trend
	Below upper-secondary education	Upper-secondary education and above	
Age 65–74 years			
No. of participants (n = 8,019)	1,849	6,170	
Incident functional disability (%)	5.9	13.7	
Incident rate/1000 person years	34.1	22.2	
Crude	1.00 (reference)	0.64 (0.57–0.71)	< 0.01
Age^c^ and sex adjusted	1.00 (reference)	0.69 (0.62–0.77)	< 0.01
Age≥75 years			
No. of participants (n = 5,830)	2,291	3,539	
Incident functional disability (%)	22.2	32.8	
Incident rate/1000 person years	101.3	89.0	
Crude	1.00 (reference)	0.87 (0.81–0.93)	< 0.01
Age^d^ and sex adjusted	1.00 (reference)	0.92 (0.86–0.99)	< 0.05

^a^ Analyses by cox proportional hazard model.

^b^ Age at leaving school.

^c^ Age (65–69, 70-74y).

^d^ Age (75–79, 80–84, or ≥85y).

We also conducted a sensitivity analysis by including participants with missing data for potential mediators (n = 13,849, **Table 2**), but the results did not change substantially. In comparison with participants with education level below upper-secondary education, the age-sex-adjusted HRs (95% CI) were 0.85 (0.80–0.90) for those with education level upper secondary education and above (*P* for trend <0.01).

### Mediators

**Table 5** shows the mediating effects of different variables. Participation in community activities had the largest effect on the relationship between education level and incident functional disability (34.7%). The corresponding mediating effects for drinking and smoking status were 3.6% and 2.0%, respectively; whereas the mediating effects for time spent walking was -6.4%.

**Table 5:** Mediation analyses of the relationship between education level [below upper secondary education (Ref.) vs. upper secondary education and above] and incident functional disability (n = 8,680)^a^, Ohsaki city, Japan, 2006–2015.

Mediator		Effect	Estimate^b^	*P* value	95% CI	
BMI		cde = nde ^c^	1.151	<0.001	1.091	1.215
≥25 vs. <25(Ref.)		nie	0.998	0.107	0.996	1.000
		total effect	1.149	<0.001	1.088	1.213
		proportion mediated (%)	n.a^d^			
Smoking status		cde = nde	1.144	<0.001	1.083	1.207
current vs. non-current (Ref.)		nie	1.003	0.029	1.000	1.005
		total effect	1.147	<0.001	1.086	1.211
		proportion mediated (%)	2.0^e^			
Drinking status		cde = nde	1.150	<0.001	1.089	1.214
current vs. non-current (Ref.)		nie	1.005	0.017	1.001	1.009
		total effect	1.156	<0.001	1.095	1.220
		proportion mediated (%)	3.6^e^			
Time spent walking		cde = nde	1.156	<0.001	1.095	1.221
<1 h/d vs. ≥1h/d (Ref.)		nie	0.992	0.001	0.987	0.997
		total effect	1.147	<0.001	1.087	1.211
		proportion mediated (%)	-6.4^e^			
Participation in the community activities		cde = nde	1.100	0.001	1.042	1.161
none vs. any (Ref.)		nie	1.048	<0.001	1.037	1.059
		total effect	1.153	<0.001	1.092	1.217
		proportion mediated (%)	34.7^e^			
Social supports		cde = nde	1.156	<0.001	1.095	1.221
none vs. any (Ref.)		nie	0.998	0.101	0.995	1.000
		total effect	1.154	<0.001	1.093	1.218
		proportion mediated (%)	n.a^d^			

CI = confidence interval; cde = controlled direct effect; nde = natural direct effect; nie = natural indirect effect.

^a^ Analyses by accelerated failure time model and logistic regression model.

^b^ Adjusted for age (65–69, 70–74, 75–79, 80–84, or ≥85y) and sex.

^c^ Exposure-mediator interactions were tested in advance but non-significant. Therefore, exposure-mediator interactions were not included in the results so that cde was equaled to nde.

^d^ Mediating effects not significant.

^e^ Proportion mediated (%) based on the regression coefficients.

### Mediators by age

**Table 6** shows the mediating effects of different potential factors among elderly aged 65–74 y. Only participation in community activities and drinking status had mediating effects (*P* value for both natural direct effect and natural indirect effect were significant, and proportion mediated were positive). The mediating effect of participation in community activities was 19.9%. Additionally, the magnitude of the mediating effect for drinking status was 3.6%; while time spent walking had a negative mediating effect which was -5.0%.

**Table 6:** Mediation analyses of the relationship between education level [below upper secondary education (Ref.) vs. upper secondary education and above] and incident functional disability, for participants aged 65–74 y (n = 5,338)^a^, Ohsaki city, Japan, 2006–2015.

Mediator		Effect	Estimate^b^	P value	95% CI	
BMI		cde = nde^c^	1.304	<0.001	1.204	1.414
≥25 vs.<25(Ref.)		nie	0.999	0.475	0.996	1.002
		total effect	1.303	<0.001	1.202	1.412
		proportion mediated (%)	n.a^d^			
Smoking status		cde = nde	1.293	<0.001	1.193	1.401
current vs.non-current (Ref.)		nie	1.004	0.054	1.000	1.008
		total effect	1.298	<0.001	1.198	1.407
		proportion mediated (%)	n.a^d^			
Drinking status		cde = nde	1.294	<0.001	1.194	1.403
current vs.non-current (Ref.)		nie	1.007	0.050	1.000	1.015
		total effect	1.304	<0.001	1.203	1.413
		proportion mediated (%)	3.6^e^			
Time spent walking		cde = nde	1.317	<0.001	1.215	1.427
<1 h/d vs.≥1h/d (Ref.)		nie	0.989	0.006	0.981	0.997
		total effect	1.301	<0.001	1.201	1.410
		proportion mediated (%)	-5.0^e^			
Participation in the community activities		cde = nde	1.238	<0.001	1.142	1.342
none vs.any (Ref.)		nie	1.048	<0.001	1.032	1.063
		total effect	1.297	<0.001	1.197	1.405
		proportion mediated (%)	19.9^e^			
Social supports		cde = nde	1.305	<0.001	1.204	1.414
none vs.any (Ref.)		nie	1.001	0.824	0.996	1.005
		total effect	1.305	<0.001	1.204	1.415
		proportion mediated (%)	n.a^d^			

CI = confidence interval; cde = controlled direct effect; nde = natural direct effect; nie = natural indirect effect.

^a^ Analyses by accelerated failure time model and logistic regression model.

^b^ Adjusted for age (65–69 and 70-74y) and sex.

^c^ Exposure-mediator interactions were tested in advance but non-significant. Therefore, exposure-mediator interactions were not included in the results so that cde was equaled to nde.

^d^ Mediating effects not significant.

^e^ Proportion mediated (%) based on the regression coefficients.

**Table 7** shows the mediating effects among elderly aged ≥75 years. Except for Participation in the community activities (mediating effect 62.5%), no mediating effect was observed due to non-significant *P* values for natural indirect effect with all potential mediators among elderly aged ≥75 years.

**Table 7:** Mediation analyses of the relationship between education level [below upper secondary education (Ref.) vs. upper secondary education and above] and incident functional disability, for participants aged ≥75 years (n = 3,342)^a^, Ohsaki city, Japan, 2006–2015.

Mediator		Effect	Estimate^b^	P value	95% CI	
BMI		cde = nde^c^	1.069	0.059	0.997	1.146
≥25 vs. <25(Ref.)		nie	0.997	0.159	0.994	1.001
		total effect	1.067	0.070	0.995	1.144
		proportion mediated (%)	n.a^d^			
Smoking status		cde = nde	1.065	0.078	0.993	1.141
current vs. non-current (Ref.)		nie	1.001	0.288	0.999	1.003
		total effect	1.066	0.073	0.994	1.143
		proportion mediated (%)	n.a^d^			
Drinking status		cde = nde	1.071	0.054	0.999	1.148
current vs. non-current (Ref.)		nie	1.001	0.427	0.998	1.005
		total effect	1.072	0.050	1.000	1.150
		proportion mediated (%)	n.a^d^			
Time spent walking		cde = nde	1.072	0.050	1.000	1.149
<1 h/d vs. ≥1h/d (Ref.)		nie	0.997	0.262	0.992	1.002
		total effect	1.069	0.061	0.997	1.146
		proportion mediated (%)	n.a^d^			
Participation in the community activities		cde = nde	1.028	0.438	0.959	1.102
none vs. any (Ref.)		nie	1.045	<0.001	1.030	1.060
		total effect	1.075	0.044	1.002	1.152
		proportion mediated (%)	62.5^e^			
Social supports		cde = nde	1.073	0.048	1.001	1.151
none vs. any (Ref.)		nie	0.997	0.100	0.993	1.001
		total effect	1.069	0.059	0.997	1.147
		proportion mediated (%)	n.a^d^			

CI = confidence interval; cde = controlled direct effect; nde = natural direct effect; nie = natural indirect effect.

^a^ Analyses by accelerated failure time model and logistic regression model.

^b^ Adjusted for age (75–79, 80–84, or ≥85y) and sex.

^c^ Exposure-mediator interactions were tested in advance but non-significant. Therefore, exposure-mediator interactions were not included in the results so that cde was equaled to nde.

^d^ Mediating effects not significant.

^e^ Proportion mediated (%) based on the regression coefficients.

## Discussion

In this cohort study, we found that education level was inversely associated with the risk of incident functional disability, in accord with previous studies [4–7, 9]. Furthermore, our results suggested that participation in community activities had a stronger effect on the above relationship than drinking and smoking status. To our knowledge, this is the first study to have evaluated mediating effects between education level and disability in a Japanese population aged 65 years and older.

In view of the possible bias caused by excluding subjects with missing data for mediators, sensitivity analyses were undertaken using 13,849 respondents including those for whom such data were missing. However, after adjustment for age and sex, the inverse relationship between education level and incident functional disability did not change substantially. Therefore, our findings with respect to the relationship between education level and incident functional disability are unlikely to be attributable to selection bias.

We used an accelerated failure time model and a logistic regression model to estimate the natural direct effect, natural indirect effect, and total effect. A “proportion mediated” was calculated as the ratio of the natural indirect effect to the total effect. We observed that participation in community activities contributed moderately (34.7%) to the relationship between education level and incident functional disability. In a prior study that analyzed mediators in the relationship between education and health in terms of disability, those explained by social participation ranged from 27.4% to 41.8% in the middle-higher education group, which was similar to our present results [15].

By contrast, the present study found that smoking status and drinking status did not have a large impact on the relationship between education level and incident functional disability. A previous longitudinal study found that this relationship was slightly mediated by smoking and drinking status [15], in line with our study.

The present study also revealed that participation in community activities had a major mediating effect among both those aged 65–74 years and those aged ≥75 years. However, we consider the mediating effect negligible among the elderly aged ≥75 years. Even though the “proportion mediated” was relatively large, the absolute values of coefficients for the natural direct effect and natural indirect effect were too small to exert influence at a population level. This difference may have been attributable to differences in socioeconomic status among elderly of different ages. In Japan, elderly people are able to continue working if they wish, and may do so after reaching retirement age [17]. According to the Population Census of 2015 [30], 67% were still working at the age of 65 but the proportion decreased to 27% by the age of 75. On one hand, education may affect employment of the younger elderly to a greater degree because they are generally employed by enterprises and institutions, whereas older elderly tend to be engaged in agricultural work. Educated younger elderly tend to have jobs that provide a tighter social network (especially with persons other than family/relatives) and higher income. These factors would promote social participation [31] and bring more health benefits. On the other hand, younger elderly are generally healthier than older elderly. Recent studies have also suggested that educated elderly benefit more from leisure activities, especially those who are healthier [31, 32].

When further looking at the mediating effect of each item of community activities among elderly people aged 65–74 years, we observed a lager mediating effect for “sports clubs, hobby groups” than other items (See S1 Table). This may because that the mediating effect of participation in sports clubs or hobby groups not only represents the effect came from social engagement, but also partially came from physical activity in social engagement.

Interestingly, among elderly aged 65–74 years, the time spent walking has negative mediating effect. This might be interpreted that participants with higher education level were less likely to walk, as shown in the baseline characteristics. A previous study has suggested that among younger elderly people (aged 54–72 years) with higher education level, there was a negative association between employment and physical activity [33], which is in line with our findings. Therefore, it is important to encourage physical activity in the elderly population, especially among younger elderly with a higher education level.

### Study strengths and limitations

Our study had a number of strengths: 1) it is the first cohort study to have investigated the role of mediators in the association between education level and incident functional disability among Japanese elderly; 2) the study had a 9-year follow-up period with high follow-up rate (98.6%).

However, there were also several limitations: 1) data on mediators were obtained only at the baseline, and information regarding such factors might have changed during the follow-up period; 2) we might not have considered unmeasured mediators, for example working conditions and income; 3) because not all candidates applied for LTCI certification, this study may not have been completely free from detection bias. The degree of this bias remains to be verified; 4) among the 23,091 individuals who provided valid responses, 6,333 who did not provide written consent for review of their LTCI information were excluded from our analyses. These individuals were less likely to have an upper-secondary and above education, and to participate in community activities. Thus, the mediating effect in the present study might have been underestimated and may not be generalizable to the Japanese population as a whole.

## Conclusion

In conclusion, the present study has shown that higher education level was significantly associated with a lower risk of incident functional disability, and that participation in community activities impacted moderately on the association between education level and incident functional disability, especially for those aged 65 to 74 years.

## Supporting information

S1 File Appendix. (DOCX)

S1 Table Mediation analyses of the relationship between education level [below upper secondary education (Ref.) vs. upper secondary education and above] and incident functional disability, for participants aged 65-74y (n = 5,338)^a^, Ohsaki city, Japan, 2006–2015. CI = confidence interval; cde = controlled direct effect; nde = natural direct effect; nie = natural indirect effect. ^a^Analyses by accelerated failure time model and logistic regression model. ^b^Adjusted for age (65–69 and 70–74 y) and sex. ^c^Exposure-mediator interactions were tested in advance but non-significant. Therefore, exposure-mediator interactions were not included in the results so that cde was equaled to nde. ^d^Proportion mediated (%) based on the regression coefficients.
(DOCX)
