# Supplementary material for: Education level and incident functional disability in elderly Japanese: The Ohsaki Cohort 2006 study
Source: PLoS One. 2019 Mar 12;14(3):e0213386. doi: 10.1371/journal.pone.0213386 (PMC6414025; doi:10.1371/journal.pone.0213386)
Supplement: S1 File — (DOCX) [file pone.0213386.s001.docx]

**Appendix**

According to Valeri, L. and Vanderweele, T. J.’s study [1], a mediation analysis could decomposes the relationships among exposure (in the present study, education level: 0 for below upper-secondary education, 1 for upper secondary education and above), outcome (incident disability), and mediators into 1) an indirect effect of exposure on outcome that is mediated through mediator, and 2) two types of direct effects of exposure that circumvent mediator to affect outcome, with consideration of interactions between exposure and mediators:

1) controlled direct effect (i.e. how much the proportion of log-survival time would change if the mediator were controlled at uniform level in the population but the education level changed from 0 to 1); 2) natural direct effect (i.e. how much the proportion of log-survival time would change if education level change from 0 to 1, but for each individual the mediator were kept at the level it would have taken at education level 0); 3) natural indirect effect (i.e. how much the proportion of log-survival time would change if education were controlled at level 1, but the mediator were changed from the level it would take if education level = 0 to the level it would take if education level = 1); and 4) total effect (i.e. how much the proportion of log-survival time would change overall if education level change from 0 to 1). Simultaniouly, effects of confunding factors on mediator and incident disability were also adjusted. Ilustration was shown as below:

1. Valeri L, Vanderweele TJ. Mediation analysis allowing for exposure-mediator interactions and causal interpretation: theoretical assumptions and implementation with SAS and SPSS macros. Psychol Methods. 2013;18(2):137-50. doi: 10.1037/a0031034. PubMed PMID: 23379553; PubMed Central PMCID: PMCPMC3659198.
