# Supplementary material for: Education level and incident functional disability in elderly Japanese: The Ohsaki Cohort 2006 study
Source: PLoS One. 2019 Mar 12;14(3):e0213386. doi: 10.1371/journal.pone.0213386 (PMC6414025; doi:10.1371/journal.pone.0213386)
Supplement: S1 Table — CI = confidence interval; cde = controlled direct effect; nde = natural direct effect; nie = natural indirect effect. aAnalyses by accelerated failure time model and logistic regression model. bAdjusted for age (65–69 and 70–74 y) and sex. cExposure-mediator interactions were tested in advance but non-significant. Therefore, exposure-mediator interactions were not included in the results so that cde was equaled to nde. dProportion mediated (%) based on the regression coefficients. (DOCX) [file pone.0213386.s002.docx]

| **S1 Table** | | | | | | |  |
| --- | --- | --- | --- | --- | --- | --- | --- |
| Mediator | | Effect | Estimate^b^ | P value | 95% CI | |  |
| Activieties in neighborhood association | |  |  |  |  |  |  |
| Non vs Yes (Ref.) | | cde = nde^c^ | 1.288 | <0.001 | 1.188 | 1.396 |  |
|  | | nie | 1.027 | <0.001 | 1.015 | 1.039 |  |
|  | | total effect | 1.323 | <0.001 | 1.220 | 1.434 |  |
|  | | proportion mediated (%) | 10.8^d^ |  |  |  |  |
|  | |  |  |  |  |  |  |
| Sports clubs, hobby groups | |  |  |  |  |  |  |
| Non vs Yes (Ref.) | | cde = nde | 1.263 | <0.001 | 1.164 | 1.369 |  |
|  | | nie | 1.047 | <0.001 | 1.030 | 1.064 |  |
|  | | total effect | 1.322 | <0.001 | 1.219 | 1.433 |  |
|  | | proportion mediated (%) | 18.4^d^ |  |  |  |  |
|  | |  |  |  |  |  |  |
| Volunteering | |  |  |  |  |  |  |
| Non vs Yes (Ref.) | | cde = nde | 1.287 | <0.001 | 1.187 | 1.395 |  |
|  | | nie | 1.032 | <0.001 | 1.018 | 1.046 |  |
|  | | total effect | 1.328 | <0.001 | 1.224 | 1.439 |  |
|  | | proportion mediated (%) | 12.5^d^ |  |  |  |  |
|  | |  |  |  |  |  |  |
| Social gathering | |  |  |  |  |  |  |
| Non vs Yes (Ref.) | | cde = nde | 1.272 | <0.001 | 1.173 | 1.379 |  |
|  | | nie | 1.041 | <0.001 | 1.025 | 1.057 |  |
|  | | total effect | 1.324 | <0.001 | 1.221 | 1.435 |  |
|  | | proportion mediated (%) | 16.0^d^ |  |  |  |  |
| CI=confidence interval; cde= controlled direct effect; nde= natural direct effect; nie= natural indirect effect. | | | | | | | |
| ^a^ | Analyses by accelerated failure time model and logistic regression model. | | | | | | |
| ^b^ | Adjusted for age (65-69 and 70-74 y) and sex. | | | | | | |
| ^c^ | Exposure-mediator interactions were tested in advance but non-significant. Therefore, exposure-mediator interactions were not included in the results so that cde was equaled to nde. | | | | | | |
|  |  |  |  |  |  |  |  |
| ^d^ | Proportion mediated (%) based on the regression coefficients. | | | | | | |
